# Paraneoplastic Leukocytosis: A Poor Prognostic Marker in Pancreatic Adenocarcinoma

**DOI:** 10.7759/cureus.9013

**Published:** 2020-07-05

**Authors:** Sohaip Kabashneh, Hammad Ali, Layla Shanah, Ala A Alkofahi, Samer Alkassis

**Affiliations:** 1 Internal Medicine, Wayne State University/Detroit Medical Center, Detroit, USA; 2 Internal Medicine, University of Hawaii, Honolulu, USA

**Keywords:** hyperleukocytosis, paraneoplastic syndrome, pancreatic adenocarcinoma

## Abstract

Hyperleukocytosis is a rare form of paraneoplastic syndrome that has been reported in adenocarcinomas, particularly pancreatic cancer. We present an elderly man with chronic abdominal pain and weight loss for six months. On examination, he had diffuse tenderness and marked ascites. A workup with a CT scan revealed a pancreatic mass, which was confirmed to be pancreatic adenocarcinoma on biopsy. His lab work showed a significant leukocytosis. An extensive infectious workup was negative. He was not on any medications known to cause a leukocytosis; therefore, his leukocytosis was attributed to his cancer. Unfortunately, he died just a few days later. This case highlights hyperleukocytosis as a paraneoplastic syndrome that is a poor prognostic sign, and can be used as a marker for disease progression.

## Introduction

Paraneoplastic syndromes are clinical disorders that are not directly related to the physical effects of the primary or metastatic tumor [1,2]. However, they are closely associated with the malignant disease and can manifest as metabolic or degenerative symptoms because of hormonal or humoral factors produced by the tumor cells [3].

Hyperleukocytosis is a rare form of paraneoplastic syndrome that has been reported in adenocarcinomas, particularly renal, pancreatic, and lung cancers [4]. It has been defined as a white cell count greater than 50,000/mL to 100,000/mL. However, experts now agree this absolute cutoff is not necessary for diagnosis [4]. Given the rarity of this phenomenon, there are limited studies elucidating the underlying pathophysiology associated with it [5].

We present a case of paraneoplastic leukocytosis in a patient with regionally advanced pancreatic carcinoma.

## Case presentation

A 63-year-old gentleman with a past medical history of HIV, diabetes mellitus, and intravenous drug use presented to the hospital for epigastric abdominal pain and unintentional weight loss of roughly six kilograms over the previous six months. He also reported a loss of appetite, generalized weakness, and fatigue. On examination, he was in mild distress and cachectic looking. He had a temperature of 36.6°C, a heart rate of 82 beats per minute, a blood pressure of 126/70 mmHg, and an oxygen saturation of 97% on room air. He had diffuse abdominal tenderness, marked distention with shifting dullness, and decreased bowel sounds, but no hepatosplenomegaly was appreciated. The examination of his lungs, heart, and extremities was unremarkable.

Complete blood count revealed leukocytosis. Biochemical labs showed acute kidney injury. Lipase was found to be elevated (Table 1).

**Table 1:** Lab results on admission

Test	Results	Reference range
White blood cells	53.3 cells/µL	3.5-10.6
Absolute neutrophil count	52.2 cells/µL	1.5-8.0
Hemoglobin	11.2 gm/dL	13.3-17.1
Patelet	382 x 10^3^/µL	150-450 x 10^3^
Creatinine	1.66 mg/dL	0.70-1.30
Blood urea nitrogen	42 mg/dL	7-20
Potassium	5.4 mmol/L	3.6-5.2
Lipase	280 U/L	12-70

**Figure 1:**
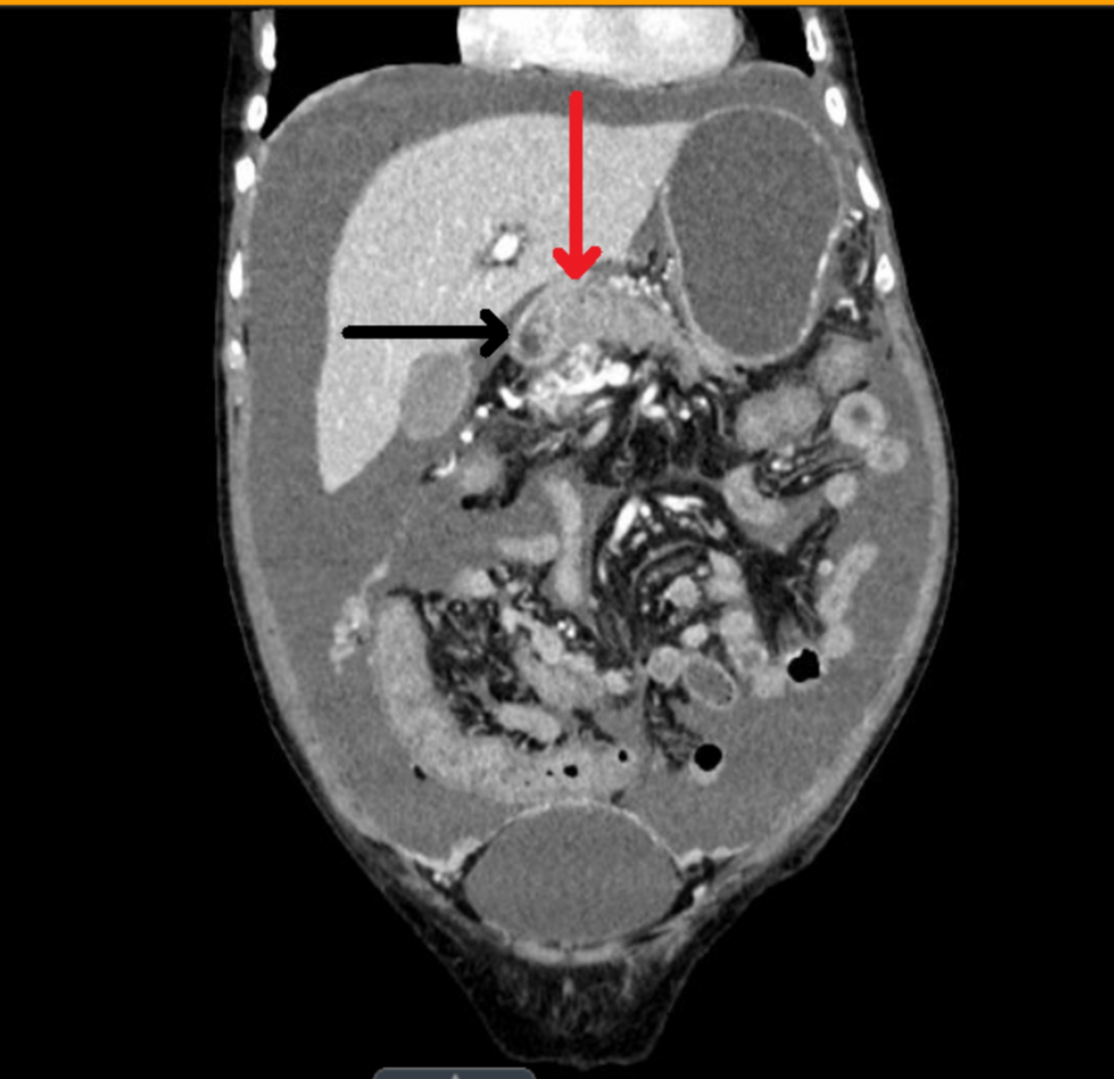
Diffuse mass-like enlargement of the pancreatic body and tail (red arrow), along with a tiny cystic area in the pancreatic tail (black arrow).

**Figure 2:**
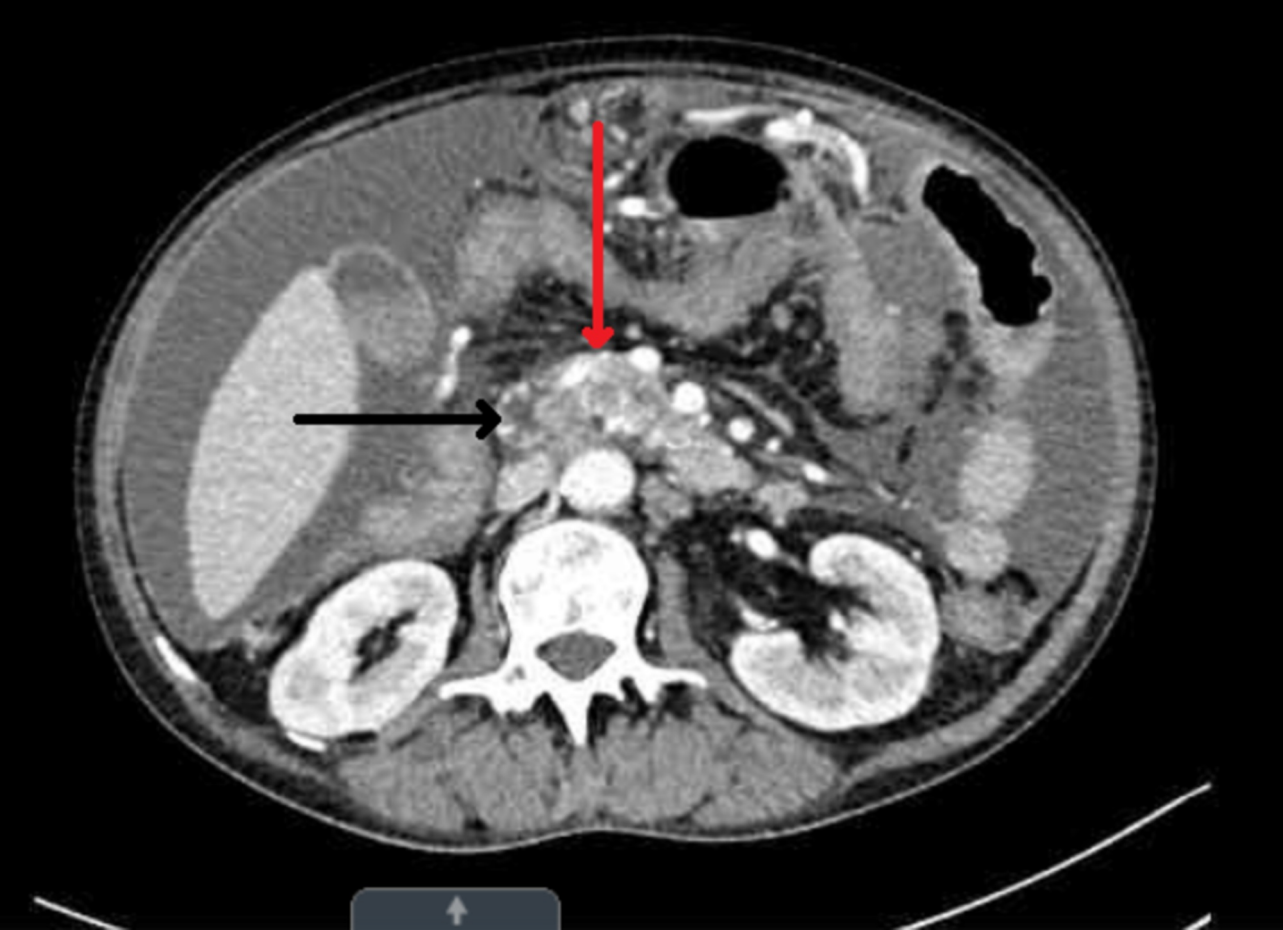
Diffuse mass-like enlargement of the pancreatic body and tail (red arrow), along with a tiny cystic area in the pancreatic tail (black arrow).

**Figure 3:**
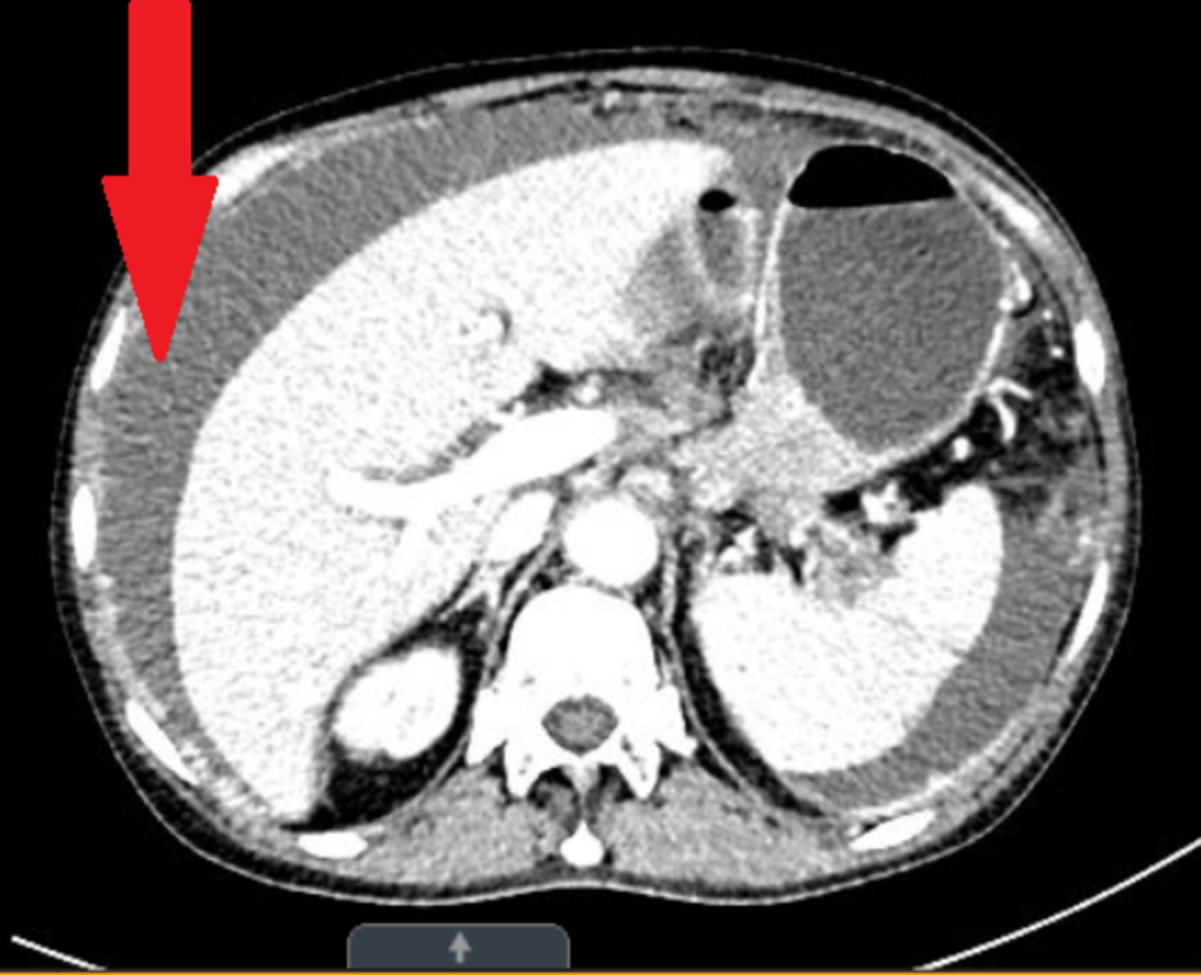
Large amounts of peritoneal fluid in the abdomen (red arrow).

CT of the abdomen was performed for abdominal pain which revealed diffuse mass-like enlargement of the pancreatic body and tail. In addition, large amounts of peritoneal fluid and multiple peritoneal masses were noted, a finding that is most consistent with metastatic pancreatic cancer (Figures 1-3).

Extensive infectious workup was done looking for an infectious etiology behind the leukocytosis, including chest X-ray, which did not reveal infiltrates. A urinalysis was clean. Multiple blood cultures were negative. Even the ascites seen on abdominal ultrasound was tapped and cultured; however, there was no growth. The patient was also tested for novel coronavirus twice and both times was negative. Despite being on empiric broad-spectrum antibiotics, whilst being worked up, his white blood cells failed to come down. Rather it was up trending to 64.5 cells/µL. The infectious disease team recommended discontinuing antibiotic therapy given negative infectious workup and no response to five days of broad-spectrum antibiotics. His leukocytosis was attributed to the advanced malignancy.

**Figure 4:**
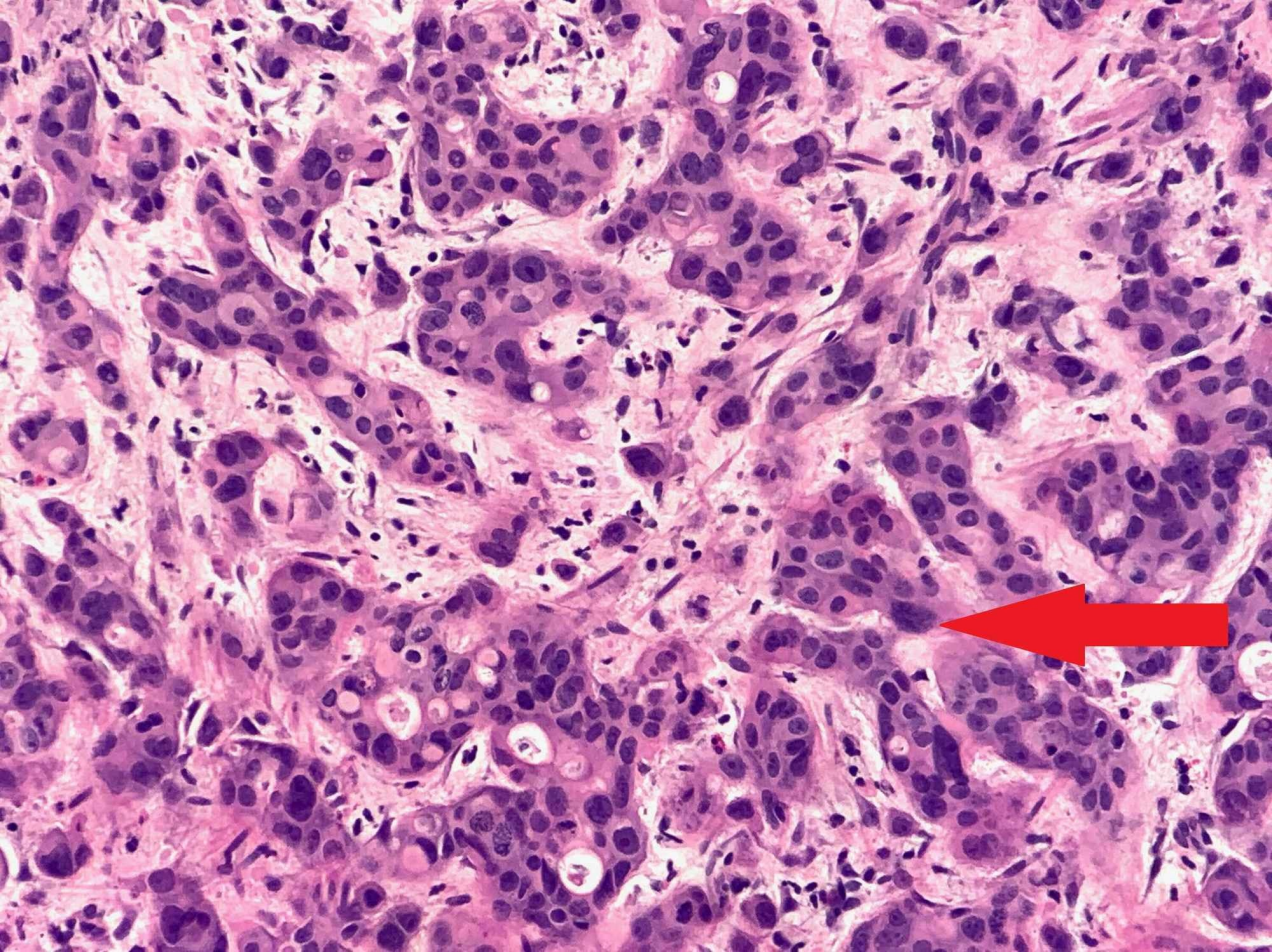
Slide showing pancreatic adenocarcinoma, malignant cells with high nucleus to cytoplasm ratio (red arrow).

Paracentesis of ascites was positive for malignant cells on cytology. There was no evidence of infection as previously mentioned. Biopsy of the pancreas done via esophagogastroduodenoscopy came back positive for pancreatic adenocarcinoma (Figure 4). Carbohydrate antigen 19-9 (CA 19-9) level came back elevated at 2,029 U/mL (normal <37).

Given the presence of local invasion and metastasis, a multidisciplinary goal of care discussion with the patient concluded with the patient choosing comfort care and hospice. Whilst awaiting placement for inpatient hospice, his condition further deteriorated and the patient died.

## Discussion

Leukocytosis can stem from infection, medications including corticosteroid, hematopoietic growth factor use, hematologic malignancy, or paraneoplastic phenomenon [5]. Once an acute infection or a medication as a cause of the leukocytosis has been excluded, the clinician should focus on malignancy as a direct or indirect cause. These cases can be challenging to differentiate from acute leukemias, but it should be kept in mind that leukemias would present with immature myeloid cells which is not the case for paraneoplastic hypercellularity. Peripheral smear for paraneoplastic hypercellularity would show mature polymorphonuclear cells and immature cells of granulocyte lineage [5].

There is a dearth of studies looking into the mechanisms causing this paraneoplastic leukemoid reaction. However, from what little studies we have, it has been shown that this hyperleukocytosis results from growth factors, such as granulocyte colony-stimulating factor, granulocyte-macrophage colony-stimulating factor, and interleukin-6 (IL-6), which are thought to be produced by the malignant cells [6].

Hyperleukocytosis in cancer is considered a poor prognostic marker and suggests an aggressive course of the disease with a large proportion of these patients dying soon after diagnosis, like our patient above [7-9]. It has been observed in in vivo studies that the colony-stimulating factors also enhance the growth of the malignant cells, thereby imparting a worse outcome [10]. Some have even postulated that colony-stimulating factors act as autocrine growth factors, resulting in malignancy proliferation and metastasis [11]. Treatment of the underlying malignancy can lessen the leukocytosis and progression of the malignancy can worsen it; hence, it can also be used as a marker for disease progression in such patients [5].

## Conclusions

The presence of hyperleukocytosis in cancer should alert the physician of a possible paraneoplastic cause. However, it should not preclude a workup for other possible causes. Moreover, hyperleukocytosis in this setting suggests an aggressive course of the disease. Treatment of underlying malignancy can lessen the leukocytosis and progression of the malignancy can worsen it; hence, it can be used as a marker for disease progression in such patients.
